# Site-Specific Genome Engineering in Human Pluripotent Stem Cells

**DOI:** 10.3390/ijms17071000

**Published:** 2016-06-24

**Authors:** Sylvia Merkert, Ulrich Martin

**Affiliations:** 1 Leibniz Research Laboratories for Biotechnology and Artificial Organs (LEBAO), Department of Cardiothoracic, Transplantation and Vascular Surgery, Hannover Medical School, 30625 Hannover, Germany; merkert.sylvia@mh-hannover.de; 2 Regenerative Biology and Reconstructive Therapies (REBIRTH), Cluster of Excellence, Hannover Medical School, 30625 Hannover, Germany; 3 Biomedical Research in Endstage and Obstructive Lung Disease (BREATH), Member of the German Center for Lung Research (DZL), Hannover Medical School, 30625 Hannover, Germany

**Keywords:** targeted genome engineering, human iPSCs, zinc-finger nucleases (ZFNs), transcription activator-like effector nuclease (TALEN), clustered regularly interspaced short palindromic repeat (CRISPR)/Cas9

## Abstract

The possibility to generate patient-specific induced pluripotent stem cells (iPSCs) offers an unprecedented potential of applications in clinical therapy and medical research. Human iPSCs and their differentiated derivatives are tools for diseases modelling, drug discovery, safety pharmacology, and toxicology. Moreover, they allow for the engineering of bioartificial tissue and are promising candidates for cellular therapies. For many of these applications, the ability to genetically modify pluripotent stem cells (PSCs) is indispensable, but efficient site-specific and safe technologies for genetic engineering of PSCs were developed only recently. By now, customized engineered nucleases provide excellent tools for targeted genome editing, opening new perspectives for biomedical research and cellular therapies.

## 1. Introduction

The groundbreaking technology for the generation of patient- and disease-specific human induced pluripotent stem cells (iPSC) holds great potential for basic research, drug development, and regenerative therapies. Human iPSCs can be derived from any somatic cell type by transient overexpression of a small number of transcription factors central to the pluripotent phenotype in embryonic stem cells (ESCs). Meanwhile, the generation of human iPSCs has become a standard procedure in many laboratories. Nowadays, easily accessible cell sources like blood [1,2], hair bulks [3], or urine [4] can be utilized for reprogramming and increasingly replace invasive procedures like skin biopsies for isolation of fibroblasts. Human iPSCs are almost indistinguishable from ESCs with respect to their phenotype and culture characteristics, and it is generally accepted that iPSCs represent a bona fide pluripotent cell type [5]. In contrast to ESCs, no embryos have to be destroyed during derivation of iPSCs. Another advantage of iPSC lines is the possibility of deriving these cells from patients suffering from monogenic and complex diseases, thereby providing limitless access to disease-specific differentiated cells for disease modelling, drug screening, and safety pharmacology. Alike ESCs, iPSCs show an unlimited potential for proliferation and differentiation, and recent critical advance in scalable mass production [6,7] and targeted differentiation of such cells (e.g., [8]) further facilitates industrial process development and future clinical application of regenerative products.

**Figure 1:**
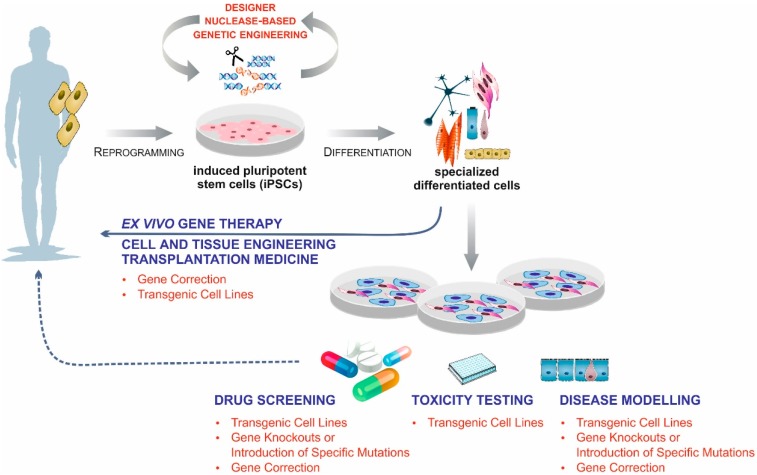
The ability to genetically modify induced pluripotent stem cells (iPSCs) is indispensable for cellular therapies and medical research. The advancements of customized engineered endonucleases provide excellent tools for the introduction of reporter and selection genes, the overexpression of therapeutic transgenes, the generation of gene knockouts, the genetic correction of mutations, or the targeted introduction of disease-specific mutations.

The development of technologies for efficient genetic modification of pluripotent stem cells (PSCs) was another critical hurdle for their use in disease modelling, drug development, and cellular therapies. In fact, various applications of genetic engineering were urgently required that would facilitate basic research and screening procedures or enable a clinical application of PSC-based cell and tissue transplants. These include (1) more efficient and targeted introduction of reporter and selection genes; (2) the controlled overexpression of therapeutic transgenes; (3) a straightforward generation of gene knockouts; (4) the genetic correction of mutations; or (5) the targeted introduction of disease-specific mutations (Figure 1).

After the development of technologies that allowed for isolation, culture, and characterisation of human ESCs, genetic modification of human PSCs remained a true challenge for many years. Efficient protocols for plasmid transfection were not available at the early phase and gene transfer was only possible using viral and especially lentiviral vectors [9]. Admittedly, such techniques did not allow for site-specific genome engineering; transgenes were lost over time, or the introduced transgenes frequently underwent silencing in undifferentiated cells or during differentiation. Later on, lipofection protocols [10] and new electroporation technologies including nucleofection [11] were developed that enabled efficient plasmid transfection [12] and the generation of human PSC lines with more stable transgene expression [13]. Although frequently less silencing is observed after stable integration of expression plasmids compared with lentivitral transduction, these approaches still rely on random integration of genetic elements and more or less unpredictable integration-site-dependent transgene expression or insertional mutagenesis.

A more specific but laborious alternative is the classic technique of gene targeting, which was well established in murine embryonic stem cells for decades [14,15]. This approach uses the homologous recombination (HR) pathway and a donor plasmid carrying a selectable transgene flanked by homologous DNA stretches of substantial length to specifically target the favoured locus. However, low targeting efficiencies [16] and challenging culture characteristics in terms of growing in colonies on a feeder-cell layer and diminished single cell survival, have prevented classic gene targeting in human PSCs from becoming broadly applicable, and positive and negative selection markers have been indispensable to identify the rare events of targeting among the typically more frequent off-target events [17,18,19].

These issues have been overcome by the introduction of customized engineered endonucleases in terms of zinc-finger nucleases (ZFNs), transcription activator-like effector nucleases (TALENs), and clustered regularly interspaced short palindromic repeat (CRISPR) RNA-guided nucleases. Since the first demonstration of ZFN-mediated gene targeting in human ESCs [20], a rapid development in genetic engineering has been accomplished. Especially the “user-friendly” CRISPR/Cas9 system has helped to advance the targeting field rapidly and enable the widespread application of this technology. Designer nucleases enable locus specific introduction of double-strand breaks (DSBs); thus, endogenous cellular DNA repair mechanisms including non-homologous end joining (NHEJ) [21,22] and HR [23,24] are utilized to efficiently introduce the intended genetic modifications nearby the induced DSB. Therefore, several publications are available addressing the details of ZFN, TALEN, and CRISPR/Cas techniques including potential off-target effects of the nucleases [25,26,27].

Meanwhile, designer nuclease technology is successfully applied by many research groups to genetically modify human PSCs, not only allowing for efficient gene inactivation through NHEJ, but also for enhanced HR-based gene targeting. In the following, we will shortly summarize the major application fields of gene targeting using designer nucleases in human PSCs to highlight the importance of this technology for cellular therapies and medical research.

## 2. Generation of Transgenic Cell Lines

The generation of transgenic cell lines carrying reporter- or selection genes represents one important application of genome editing in human PSCs. Such PSC lines are indispensable for the enrichment of distinct cell lineages after differentiation, for the monitoring of stem cells and their progenies in vitro and in vivo, and for the measurement of cell functions. Even future clinical application might depend on the safe introduction of transgenes such as suicide genes.

Generation of transgenic PSC lines was certainly possible already prior to the development of designer nucleases. However, random integration of transgenes with unpredictable expression levels and frequently observed silencing in undifferentiated cells and especially during differentiation typically aggravated the generation of useful cell lines. By applying modern genome engineering approaches, transgenic lines can be generated either by targeted integration into a so-called “safe harbour site”, applying tissue-specific or constitutive promoters, or by targeted integration into the endogenous gene of interest. Safe harbour sites can be located intragenic in introns or extragenic, and enable robust transgene expression not only in undifferentiated cells but also in the differentiated derivatives. They are considered as safe because the integration of transgenes apparently does not lead to oncogenic transformation or show any signs of genotoxic effects [28]. For the last few years, several groups have used designer nucleases for the targeted introduction of constitutively expressed fluorescence proteins in the AAVS1 or the CCR5 locus in human PSCs [20,29,30,31,32,33,34,35,36,37,38,39], as flanking genes of these loci may be unregulated [30]. In addition, the ROSA26 locus [18] and the citrate lyase beta like gene locus (CLYBL) [40] also represent promising safe harbour sites for application in human PSCs, but so far no further studies assessing the safety of these loci have been performed. AAVS1, CLYBL, and ROSA26 are transcriptionally active sites in the human genome, whereas CCR5 is a predominantly inactive site in human PSCs but not in hematopoietic cell lineages. The stable transgene expression in differentiated cells is, for instance, essential for reporter gene-based molecular imaging that allows for transplantation experiments, and Wang et al. generated a triple reporter for fluorescence- and bioluminescence imaging and positron emission tomography, whereby they could successfully track the survival of differentiated cardiomyocytes and endothelial cells in murine transplantation models [41]. Such approaches greatly accelerate the transition from basic research to clinical translation, since the applied reporter cells lines not only allow for tracking cell survival and distribution, but may allow for the visualisation of even cell differentiation and cell function in vivo. Two other groups demonstrated the possibility to control transgene expression at the AAVS1 locus by inserting a tetracycline response element [42,43]. Qian et al. further facilitated this approach by constructing an AAVS1 targeting vector for inducible transgene expression with a multiple cloning site for easy swap of a gene of interest, and could demonstrate the inducible overexpression of neurofilament-low molecular weight (*NEF-L*) gene in differentiated spinal neurons [43]. In terms of the application of tissue-specific promoters, a floxed dual-fluorescence reporter transgene was introduced into the AAVS1 locus of human ESCs, which was successfully used for the isolation of specific cell populations through the combination with certain vectors for cell-type-specific Cre recombinase expression [44]. Moreover, the strategy of integration of a doxycycline-inducible Cas9 expression cassette into the AAVS1 locus of human PSCs [45] has proven very useful for a number of gene editing approaches [46,47].

Besides the application of safe harbour loci, the positioning of reporter elements under the control of endogenous loci is highly facilitated by the use of designer nucleases. Hence, the generation of *OCT4*^EGFP^ reporter cell lines monitoring the pluripotency state of human PSC cultures [42,48,49,50,51], *PITX3*^EGFP^ reporter cell lines for monitoring the differentiation into dopaminergic neurons [42,48], as well as *LGR5*^EGFP^ reporter cells for the production of human intestinal tissue [52] have already been reported.

## 3. Gene Knockout and Introduction of Disease-Specific Mutations

The generation of human PSC knockout lines or the targeted integration of specific mutations for the establishment of isogenic disease models is definitely of great significance for medical research and drug screening, but classic gene targeting in human PSCs has been described in very few cases [17,18,19,53,54]. Only the development of designer nucleases resulting in a substantially increased frequency of targeted HR finally facilitated gene targeting in human PSCs. Meanwhile, various groups demonstrated the generation of gene knockouts for human stem-cell-based disease models, such as the HR-based knockout of the endogenous *PIG-A* gene for the generation of glycosyl phosphatidylinositol-anchored proteins (GPI-AP)-deficient iPSCs [55]. Efficient gene inactivation through error-prone NHEJ was used for the targeted disruption of the *HPRT1* gene or both alleles of the DNA methyltransferase 3B (*DNMT3B*) gene to create an isogenic disease model of the Lesch–Nyhan syndrome [56] or a human iPS cell model for immunodeficiency-centromeric region instability-facial anomalies (ICF) syndrome [57], respectively. Another group investigated the generation of mutant alleles for up to 15 different genes by NHEJ using TALENs and CRISPR/Cas9 targeting as a means of performing rigorous disease modelling [58,59]. They demonstrated the generation of multiple distinct targeted cell lines with different genetic backgrounds, and concluded, e.g., for *SORT1*, that the observed cellular phenotypes are related to gene function [58]. Comparable to these studies, several other groups reported the successful generation of knockout cell lines using TALEN and CRISPR/Cas9 technology [45,60,61,62,63,64,65,66].

For modelling of other genetic diseases, not a gene knockout, but editing of just a few nucleotides is necessary. This was, e.g., reported for the introduction of disease-associated point mutations in the *α-synclein* gene (Parkinson’s disease), the *AKT2* gene (insulin resistance), or the *APOE* gene (Alzheimer’s disease), respectively [45,58,59,67]. This also allows for studying risk variants in coding sequences or regulatory regions, as shown for Parkinson’s disease using CRISPR/Cas9-mediated gene editing [68]. For modelling of the mitochondrial cardiomyopathy of Barth syndrome, a specific mutation was introduced into the *TAZ* gene of iPSCs [69], and, for mimicking amyotrophic lateral sclerosis, another group introduced a mutation in the human *SOD1* locus [70]. In principle, scarless gene targeting is applied for the integration of specific mutations and also for gene correction. This has the advantage of almost no side effects or alterations in the genome of PSC clones, which is mandatory in order to remain as close as possible to the native genomic state for ex vivo gene therapy, drug screening, or disease modelling. In general, an accurate recapitulation of human disease is required to elucidate underlying mechanisms of pathogenesis. Here, one major advantage of the iPSC technology is the possibility of directly correlating the clinical phenotype of a certain patient with the cellular phenotype of his or her cells in vitro. Unfortunately, recent practical experience demonstrated that different iPSC clones generated from one donor show significant differences in culture behaviour and differentiation capacity. Whether this also extends to functionality of their derivatives and whether this is mainly due to epigenetic differences or due to genomic variations in individual cells acquired during lifetime is not yet clear. As a consequence, it is mandatory to analyse several iPSC clones and their functional derivatives, and to carefully evaluate resulting data.

## 4. Gene Correction

To date, gene correction in human iPSCs using designer nucleases has been accomplished from several monogenetic diseases either by genotypic correction of the underlying defective endogenous gene or by insertion of a functional gene into a safe harbour locus. The functional phenotypic correction via targeted insertion of the respective genes into the AAVS1 locus in disease-specific iPSCs was reported, e.g., for human X-linked chronic granulomatous disease (X-CGD) and α-thalassemia [71,72,73]. For both diseases, the functional correction of the appropriate phenotypes was achieved as demonstrated by restored oxidase activity in differentiated granulocytes and chain balance in differentiated erythroid cells, respectively.

Direct genetic correction of disease related mutations by nuclease-based HR is often more difficult, but can be accomplished with or without leaving footprints in the genome. Our own group demonstrated the feasibility of footprintless targeting without any selection through targeted insertion of a novel restriction site into the AAVS1 locus using TALEN technology and a single stranded oligonucleotide [37]. Meanwhile, several groups reported on the genotypic correction of the *β-globin* gene in sickle cell disease iPSCs [74,75,76,77,78]. By applying different targeting strategies, partial or even complete restoration of the *β-globin* expression in differentiated erythrocytes could be achieved. Moreover, differentiation into hepatocyte-like cells with restored cellular function in vitro and even in vivo was demonstrated after the genotypic correction of the *α1-antitrypsin* (*A1AT*) gene in *A1AT1*-deficient patient iPSCs [79,80]. Additionally, for β-thalassemia [81,82], muscular dystrophy [83] and haemophilia [84] gene correction in patient-specific iPSCs using CRISPR/Cas9 have been reported. For cystic fibrosis iPSCs, correction of the underlying F508del mutation gave rise to the restoration of the chloride channel function in differentiated epithelial cells [85]. Notably, the endogenous gene correction of a splice site mutation in X-linked severe combined immunodeficiency (SCID-X1) patient iPSCs using TALENs [86] highlights the potential of this genome editing technology for the development of alternative therapeutic options, especially regarding the leukemogenic concern of viral gene therapy for SCID-X1 patients [87].

## 5. Current Limitations

Regardless of the substantial progress of targeted gene editing in human PSCs with designer nucleases, there are still key issues that have to be solved. One major issue is the high clonal diversity of human PSC lines, which results in big differences concerning transfection efficiency and single cell cloning and thus targeting efficacy. Some PSC lines will be more difficult to handle and to target than others, so protocols have to be adjusted consistently to individual cell clones. This is especially true for unique patient-specific iPSC lines but is of course not required if universal human PSC lines with established targeting protocols are applied for different gene targeting approaches. Nevertheless, some loci will be difficult to access at all, due to their chromatin status or influences of the surrounding genetic context. However, the most important question that remains so far is certainly the influence of off-target activities of the nucleases, especially with regard to cell therapies. Notably, for the CRISPR/Cas9 system, there is continuous improvement of the safety of designer nucleases, but further studies are required to estimate off-target activities for all types of designer nucleases. However, reliable biological read-outs for the real impact on cell function and mutagenesis are still missing, but may be necessary for broad clinical application.

In terms of the application of the iPSC technology and designer nucleases for disease modelling and drug screening, the question of the most adequate controls is still controversial. Initially, cells from healthy family members were considered as adequate controls, but such cells are not isogenic and even if family members share a substantial proportion of their genome with the respective patient, genetic modifiers can significantly influence the cellular disease phenotype. Current genome engineering technologies now offer the opportunity to generate controls, which have, at least in theory, exactly the same genomic background as the corresponding diseased iPSC clone. Nevertheless, similar to differences between primary iPSC clones, gene editing with the inevitable selection of correctly targeted single cell clones apparently leads to phenotypic differences that have to be considered.

## 6. Conclusions

The groundbreaking iPSC technology allows for the production of sufficient patient-derived cell material for basic biomedical research, drug development, and future cellular therapies. The value of iPSCs is now substantially further extended through recent advances in highly efficient and sequence-specific genome engineering approaches. Reporter and selection genes can be integrated at specific sites of the iPSC genome leading to well defined transgene expression levels or cell type specific expression, which substantially facilitates improvement of differentiation protocols, cell enrichment, monitoring of transplanted cells in vivo, as well as in vitro disease modelling, and drug screening. Moreover, even footprintless correction or insertion of disease-specific single nucleotide mutations is possible, which may not only simplify in vitro assays but further enables the safe introduction of selection and suicide genes, or of therapeutic transgenes. It is foreseeable that the combination of the two groundbreaking developments, iPSCs and targeted genome engineering using designer nucleases, will revolutionize medical research and drug development. In addition, both technologies will provide the basis for future development of a large bundle of innovative regenerative therapies.

## Abbreviations

iPSC: induced pluripotent stem cell
PSC: pluripotent stem cell
ESC: embryonic stem cell
HR: homologous recombination
DSB: double strand break
NHEJ: non-homologous-end-joining
ZFN: zinc-finger nuclease
TALEN: transcription activator-like effector nuclease
CRISPR: clustered regularly interspaced short palindromic repeat
